# The Role of Non-LTR Retrotransposons in Sterile Inflammation: Mechanisms and Therapeutic Potential

**DOI:** 10.3390/biomedicines14020272

**Published:** 2026-01-26

**Authors:** Hua Yang, Xin Chen, Tamara Saksida, Melita Vidaković, Sizhuo Chen, Vuk Savkovic, Mingyue Chen, Shaobin Wang, Zhenhuan Zhao

**Affiliations:** 1 Key Laboratory of Fermentation Engineering (Ministry of Education), Hubei University of Technology, Wuhan 430068, China; 2 National “111” Center for Cellular Regulation and Molecular Pharmaceutics, School of Life and Health Sciences, Hubei University of Technology, Wuhan 430068, China; 3 Department of Immunology, Institute for Biological Research “Siniša Stanković”—National Institute of the Republic of Serbia, University of Belgrade, 11060 Belgrade, Serbia; 4 Department of Molecular Biology, Institute for Biological Research “Siniša Stanković”—National Institute of the Republic of Serbia, University of Belgrade, 11060 Belgrade, Serbia; melita@ibiss.bg.ac.rs; 5 Department of Cranial Maxillofacial Plastic Surgery, University Hospital Leipzig, 04103 Leipzig, Germany; 6 Department of Ophthalmology, University of Virginia, Charlottesville, VA 22903, USA; 7 Center for Advanced Vision Science, University of Virginia, Charlottesville, VA 22903, USA

**Keywords:** non-LTR retrotransposons, sterile inflammation, LINEs, SINEs, mechanisms, therapeutic potential

## Abstract

Non-long terminal repeat (Non-LTR) retrotransposons are mobile genetic elements that replicate through a “copy-and-paste” mechanism, enabling their expansion within the genome. Aberrant activation of these elements can induce genomic instability, elicit cellular stress responses, and activate inflammasome signaling, leading to tissue injury and disease. The central process of sterile inflammation involves the release and recognition of damage-associated molecular patterns (DAMPs), endogenous molecules that initiate inflammatory responses and form a common basis for many sterile inflammatory disorders. Recent studies have identified non-LTR retrotransposons as key endogenous triggers of DAMP-like signaling that drive sterile inflammation in both neuronal and non-neuronal tissues, contributing to the development of neurodegenerative and other chronic inflammatory diseases. In this review, we summarize recent advances in understanding how non-LTR retrotransposons, particularly LINE and SINE elements, influence sterile inflammation and disease pathogenesis. We highlight how their mobilization reshapes genomic architecture and gene regulation, and how the resulting signaling cascades promote chronic inflammation, immune dysregulation, and tissue injury. We also discuss emerging therapeutic strategies aimed at suppressing retrotransposon activity or interrupting downstream inflammatory signaling for treating sterile inflammation-related diseases.

## 1. Introduction

Non-LTR retrotransposons are intrinsic components of mammalian genomes, with long interspersed nuclear elements (LINEs, particularly LINE-1) and short interspersed nuclear elements (SINEs, such as the human Alu element) constituting the vast majority [1]. LINEs possess autonomous mobility due to their coding for essential retrotransposition proteins such as reverse transcriptase and nucleases; SINEs typically lack coding capacity and must “hijack” the protein machinery of LINEs for propagation [2]. Under normal physiological conditions, these elements are strictly silenced through epigenetic mechanisms such as DNA methylation and histone modifications. However, in pathological states like cellular aging, genotoxic stress, metabolic disorders, or epigenetic dysregulation, their transcriptional suppression mechanisms are lifted [3]. The abnormally expressed RNA intermediates or cDNA produced through reverse transcription transform from “selfish elements” into endogenous danger signals, forming a direct molecular bridge linking genomic instability to sterile inflammatory responses [4].

Aseptic inflammation refers to an inflammatory response triggered by damage-associated molecular patterns (DAMPs) within the body or exogenous non-biological stimuli without the direct involvement of viable pathogens [5]. Although the triggers are non-infectious, the activated innate immune response pathways highly overlap with those of infectious inflammation, ultimately leading to the release of potent pro-inflammatory cytokines like interleukin-1β, interleukin-18, and type I interferons [6]. This persistent, low-grade chronic inflammatory state constitutes a core pathological feature of atherosclerosis, neurodegenerative diseases, autoimmune disorders, metabolic syndrome, and aging itself [7]. Therefore, identifying the sources and activation mechanisms of endogenous DAMPs is crucial for understanding and intervening in these diseases [8].

Recent studies have revealed two core and interrelated molecular pathways through which LINE and SINE elements activate sterile inflammation. The first pathway centers on cytoplasmic nucleic acid sensing: in senescent or stressed cells, LINE-1 cDNA can be recognized by cGAS, activating the STING/type I pathway to drive a type I interferon response [9]. Simultaneously, its double-stranded RNA intermediates can also be detected by MDA5, triggering a similar interferon response. This is considered a key driver of the senescence-associated secretory phenotype [10]. The second pathway focuses on direct inflammasome activation: SINE RNAs accumulate in the cytoplasm, where they are recognized by specific RNA-binding proteins. This facilitates the non-canonical assembly and activation of NLRP3 and NLRC4 inflammasomes, leading to Caspase-1 cleavage and the mature release of IL-1β/IL-18. Additionally, the LINE-1 ORF2 protein may indirectly promote DAMP release by disrupting cell membrane integrity, thereby amplifying inflammatory signaling [11]. These mechanisms have been experimentally validated across diverse pathological models, ranging from age-related macular degeneration to autoimmune diseases [12].

The role of non-LTR retrotransposons in sterile inflammation has evolved from a marginal concern to a leading hotspot [13]. However, significant uncertainties remain regarding the upstream cellular signaling networks that activate them, the cross-talk and cascade amplification effects between different immune pathways, and their tissue-specific and disease microenvironment-specific regulation [14]. This review aims to systematically map the complete process of non-LTR retrotransposons in sterile inflammation. We begin by examining their structure and fundamental functions, elucidating how LINE and SINE elements undergo retrotransposition through reverse transcription and influence host gene expression. We then explore how these abnormally activated “genomic elements” transform into “danger signals” that trigger inflammation. We will focus on detailing the specific mechanisms by which their RNA or cDNA products are recognized by the immune system and how these mechanisms function in sterile inflammatory diseases such as neurodegeneration and autoimmunity. Finally, based on this, we will evaluate the therapeutic potential and challenges of targeting this pathway, offering new therapeutic approaches for related diseases.

## 2. Structure and Function of Non-LTR Retrotransposons

LINEs have complex structures and are typically between 3000 and 7000 base pairs in length [15]. They are a type of autonomous retrotransposon that can independently encode the enzymes required for retrotransposition [16]. Their structure mainly includes a 5′ untranslated region (5′UTR), which contains promoter elements that drive the transcription of the retrotransposon itself [17]: one or more open reading frames (ORFs), include at least one ORF encoding reverse transcriptase and nucleic acid endonuclease, which are essential for the replication and integration of retrotransposons, as well as a 3′ untranslated region (3′UTR) and poly(A) tail, which contribute to the stability of retrotransposon RNA and retrotransposition efficiency [18]. Transposable elements have had a profound impact on the structure and function of genomes [19]. On the one hand, their insertion can cause gene mutations, such as insertion into gene coding regions or regulatory regions, which may affect the normal expression and function of genes, thereby triggering genetic diseases. On the other hand, LINE retrotransposition also promotes genome evolution by introducing new sequences into the genome, providing raw materials for genome diversity and adaptive evolution [20]. In addition, LINEs can also carry other DNA fragments from the host genome to new locations through retrotransposition, which is significant in the formation of new genes and genome recombination [21].

SINEs are relatively short, typically ranging in length from 100 to 300 base pairs, and never exceeding 600 base pairs [22]. These non-autonomous retrotransposons do not encode the enzymes required for retrotransposition themselves, but rely on reverse transcriptases provided by LINEs to complete the retrotransposition process [23]. The structure of SINEs typically includes an RNA polymerase III promoter, which can produce high-copy transcripts, giving them high retrotranspositional activity in the genome [24]. Their insertion may cause gene mutations, alter gene expression patterns, and even form new gene regulatory elements [25]. For example, the insertion of SINEs may activate or inhibit the expression of neighboring genes, thereby affecting the spatiotemporal expression pattern of genes [26].

## 3. The Retrotransposition Processes of LINEs and SINEs Both Follow a “Copy and Paste” Mechanism

**Figure 1:**
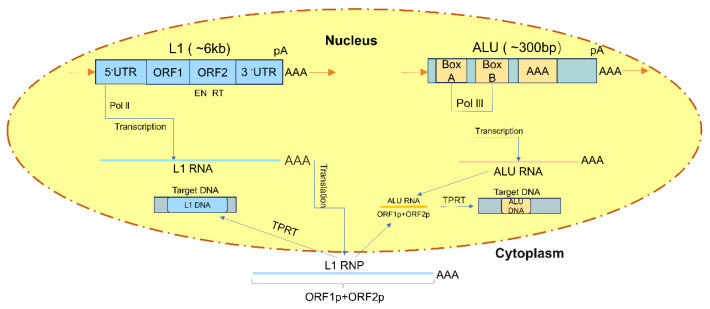
The retrotransposition process of LINE-1 and Alu [31]. EN: endonuclease, RT: reverse transcriptase. LINE-1 is transcribed into LINE-1 RNA by Pol II, and Alu is transcribed into Alu RNA by Pol III. LINE-1 RNA is translated into proteins ORF1p and ORF2p, forming LINE-1 RNP. The ORF2p protein, through its endonuclease and reverse transcriptase activities, collaboratively completes the reverse transcription process initiated by the target sequence, thereby inserting LINE-1 into new genomic sites. Alu is transcribed into Alu RNA by Pol III, which then binds to LINE-1 RNP and inserts Alu into new sites through the TPRT process.

This process involves the transcription of retrotransposon RNA, reverse transcription into cDNA, and integration of cDNA into the host genome [27]. The retrotransposition process of LINEs involves three main steps: transcription, reverse transcription, and integration [28]. First, LINEs use their own promoter elements for transcription to produce RNA transcripts. Subsequently, these RNA transcripts are reverse transcribed into cDNA by their own encoded reverse transcriptase in the cell nucleus. Finally, the cDNA is integrated into a new location in the host genome through the action of nucleic acid endonucleases [29]. During retrotransposition, the target site in the host genome is temporarily replicated, forming a target site duplication (TSD) [30]. Notably, the retrotransposition of LINEs and SINEs relies on “target-primed reverse transcription” (TPRT): LINEs first use their own encoded ORF2 protein to cleave the target DNA single strand within the nucleus, exposing a free 3′-OH end. This end then serves as a primer for direct in situ reverse transcription of the first strand—without requiring integrase or LTRs. The second strand is completed by host repair enzymes, often resulting in 5′-truncation and poly(A) tailing. The retrotransposition process of SINEs is similar to that of LINEs, but they require the reverse transcriptase of LINEs to complete the reverse transcription of RNA transcripts and the integration of cDNA (Figure 1) [31]. Utilize the ORF2 protein provided by LINEs to accomplish the same TPRT process [32]. At the same time, the retrotransposition activity of LINEs and SINEs is regulated by various factors, including epigenetic modifications of the host genome and the binding of transcription factors [33,34]. For example, certain epigenetic modifications such as DNA methylation and histone modifications can suppress the retrotransposon’s retrotransposition activity, while specific transcription factors may bind to the promoter region of the retrotransposon, promoting its transcription and retrotransposition [35].

## 4. The Role of LINEs and SINEs in Gene Expression Regulation

LINEs and SINEs play multifaceted roles in gene expression regulation [36]. For LINEs, their transcriptional activity can influence gene expression [37]. Studies have shown that transcriptionally active LINEs can enrich RNA polymerase II (Pol II), forming chromatin topological domains (TAD) boundaries, thereby regulating gene expression [38]. In addition, LINEs can activate or inhibit the expression of neighboring genes through interactions with transcription factors [39]. For example, the insertion of certain LINEs may alter transcription factor binding sites, thereby affecting gene transcriptional activity [40]. For SINEs, their insertion can alter gene expression patterns [41]. For example, SINE insertion can lead to changes in epigenetic modifications in the gene promoter region, thereby affecting gene transcriptional activity [42].

The retrotranspositional activity of SINEs can also indirectly regulate gene expression by affecting chromatin structure and the three-dimensional structure of the genome [43]. These evidences explain how LINEs and SINEs, as non-LTR retrotransposons, have a profound impact on genome structure and gene expression regulation [44]. The dynamic behavior and regulatory mechanisms of these retrotransposons provide important insights into the evolution of the genome and the regulation of gene expression, and also offer new ideas for research into the pathogenesis of related diseases and their treatment [45].

## 5. The Role of LINEs and SINEs in Sterile Inflammatory Diseases

Non-infectious inflammatory diseases include rheumatoid arthritis (RA), systemic lupus erythematosus (SLE), atherosclerosis, etc. [46]. These diseases are a group of chronic conditions characterized by inflammation-mediated immune abnormalities that affect multiple tissues and organs and exhibit high heterogeneity [47].

Recent studies have shown that LINE and SINE retrotransposons play important physiological and pathological roles in sterile inflammatory diseases [48]. In systemic lupus erythematosus, studies have shown that LINE-1 exhibits hypomethylation and high expression. By analyzing mixed RNA from kidney tissue of SLE patients, minor salivary glands (MSGs) of primary Sjögren’s syndrome (SS) patients, and healthy controls’ MSGs, it was found that the relative expression of LINE-1 transcripts in diseased tissues was significantly higher. IFN-I levels also gradually increase. Since the production of IFN-I is a characteristic of both SLE and SS, this further drives the pathological progression of SLE [49]. In a model of Aicardi–Goutières syndrome (AGS) with impaired function of triple-repaired nucleic acid exonuclease I (TREX1), the absence of TREX1 prevents it from cleaving nucleic acids in the cytoplasm, leading to nucleic acid accumulation. Research has found that LINE-1 retrotransposons are the primary source of cytoplasmic nucleic acid accumulation, which subsequently triggers a type I interferon-related inflammatory response [50]. In RA, LINE-1 drives synovial fibroblasts toward an invasive-inflammatory positive feedback loop through a four-step cascade of “low methylation-transcription activation-protein expression-immune amplification,” serving as a key molecular switch linking epigenetic dysregulation to joint destruction [51]. In age-related macular degeneration, the loss of DICER1 (an RNase III enzyme) leads to the accumulation of Alu RNA, which can directly induce the death of retinal pigment epithelial (RPE) cells, accelerating the development of a late-stage form of age-related macular degeneration known as geographic atrophy (GA), which can result in bilateral blindness [52]. In human inflammatory bowel disease, Adenine-to-inosine (A-to-I) editing is a very common post-transcriptional modification of RNA, mediated by the adenine deaminase acting on RNA (ADAR) enzyme, elevated levels of endogenous Alu dsRNA and reduced levels of endogenous Alu RNA A-to-I editing promote innate immune responses and result in severe pathological consequences [53]. In atherosclerotic diseases, Alu elements regulate distal genes, leading to increased cell proliferation and adhesion as well as reduced cell apoptosis, all of which are direct manifestations of atherosclerosis [54]. In studies on the abnormal activation of the innate immune receptor MDA5 in a series of immune diseases, Alu RNA can act as its ligand to cause abnormal activation, leading to an imbalance in immune regulation and resulting in sterile inflammatory diseases [55]. In addition, studies on aseptic inflammatory responses have found that RNA helicase DDX17 can promote SINE RNA activation of the NLRC4 inflammasome, leading to the formation of inflammation. Inhibiting DDX17-mediated inflammasome activation can significantly reduce IL-1β secretion, providing a new target for disease treatment [10]. In multiple sclerosis (MS), Alu dsRNA that has not undergone A-to-I RNA editing is an effective activator of pro-inflammatory transcriptional responses, leading to increased activation of downstream inflammatory responses. It also induces the production of interferon-stimulated genes (ISGs) through increased levels of endogenous Alu dsRNA, and increased ISG expression is also a hallmark of many human autoimmune diseases [56].

## 6. LINEs and SINEs Retrotransposons Trigger Sterile Inflammation Through Nucleic Acid Sensing Pathways and Self-Antigens

First, in terms of DNA sensing pathways, active retrotransposition of retrotransposons produces a large number of abnormal DNA molecules, including cDNA produced by LINE-1 reverse transcription and DNA damage fragments caused by genomic insertion [57]. These abnormal DNA sequences are recognized by cGAS in the cytoplasm, which catalyzes the synthesis of the second messenger cGAMP, thereby activating the STING-TBK1-IRF3 signaling cascade [58]. The sustained activation of this pathway leads to the excessive production of type I interferons and proinflammatory cytokines, resulting in a distinct interferon signature gene expression profile in patients with SLE, and also plays an important role in promoting age-related inflammation [59]. The abnormal activation of this DNA sensing mechanism is an important molecular basis for maintaining chronic inflammation [60]. It indicates that during cellular senescence, LINE-1 retrotransposons undergo transcriptional de-repression. The cytoplasmic cDNA produced by their transcription activates the cGAS-STING pathway, leading to the secretion of type I interferons and senescence-associated secretory phenotype (SASP), thereby driving inflammaging [61].

In the RNA sensing pathway, special RNA structures produced by retrotransposon transcription play a key role [62]. Notably, the innate immune activation and inflammatory responses driven by retrotransposons can be categorized into two distinct mechanisms based on whether they depend on new genomic insertion events [63]. The first is the classical, transposon-dependent mechanism, wherein DNA sensors (such as cGAS) are activated through the generation of new cDNA insertions. The second is a transposon-independent mechanism: highly expressed LINE-1 RNA and its encoded ORF1 protein (even from truncated or non-functional transcripts), along with accumulated Alu-derived double-stranded RNA, can themselves be recognized by cytoplasmic RNA sensors (such as RIG-I, MDA5) or DNA sensors, respectively, thereby inducing robust type I interferon responses and inflammation. In human diseases, the latter mechanism often plays a more prevalent and critical role [64]. The double-stranded RNA formed by the RNA transcript of LINE-1 and the Alu element can be specifically recognized by pattern recognition receptors such as RIG-I and MDA5. This recognition activates downstream signaling through the MAVS adapter protein, ultimately leading to the activation of the transcription factors IRF3 and NF-κB [65]. It is worth noting that this RNA sensing mechanism can be achieved both through Toll-like receptor (TLR) dependent pathways and through TLR-independent pathways such as RIG-I/MDA5 [66]. In diseases such as RA, the sustained activation of this RNA sensing pathway promotes the excessive production of interferons and various inflammatory factors, providing a continuous stimulus signal for the formation of a chronic inflammatory microenvironment [67].

Activation of NLRP3 inflammasomes is the third important mechanism of retrotransposon-induced inflammatory responses [68]. Retrotransposons can activate this inflammasome in several unique ways, such as Alu RNA promoting mitochondrial ROS production, indirectly facilitating NLRP3 inflammasome assembly [69]. Additionally, Alu double-stranded RNA is recognized by the Z-RNA sensor ZBP1, which then recruits RIPK3/CASP8 to form the ZBP1-PANoptosome. This complex directly catalyzes NLRP3-ASC speckle nucleation and CASP1 self-cleavage while simultaneously initiating the PANoptosis cascade, a triple cell death pathway encompassing pyroptosis, apoptosis, and necrotic apoptosis. This process converts retrotransposition activity into potent inflammatory signaling output [70]. These different pathways ultimately converge on the activation of caspase-1, promoting the maturation and release of potent inflammatory factors such as IL-1β and IL-18 [71,72]. In atherosclerotic plaques and synovial tissue in RA, abnormal activation of this pathway is closely related to the severity of local inflammatory responses [73].

**Figure 2:**
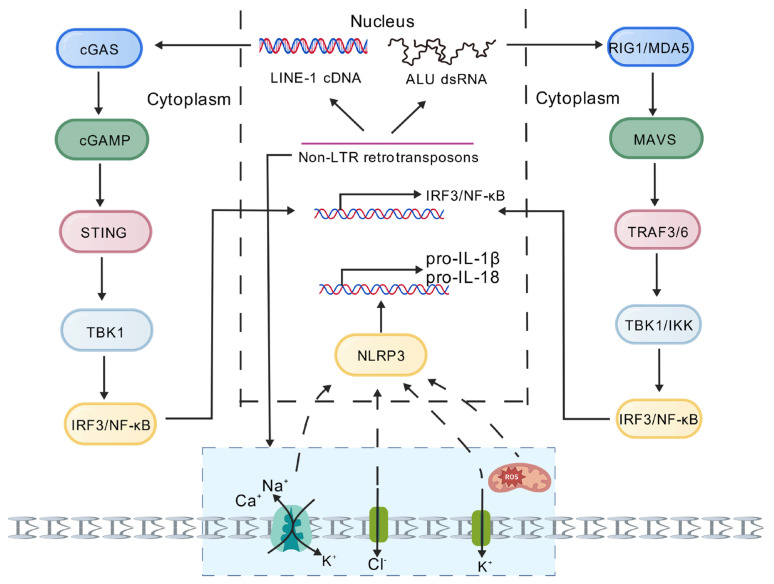
Schematic diagram of the innate immune response mechanism mediated by IRF3/NF-κB and NLRP3 inflammasome activated by non-LTR retrotransposons through the cGAS-STING and RIG-I/MDA5-MAVS pathways [75]. LINE-1 cDNA activates the cGAS-cGAMP-STING-TBK1 axis, while Alu dsRNA activates the RIG-I/MDA5-MAVS-TRAF3/6-TBK1/IKK axis, leading to the co-phosphorylation of IRF3/NF-κB and upregulation of pro-IL-1β/18. Na^+^ influx, K^+^/Cl^−^ efflux, and Mitochondrial reactive oxygen species (mtROS) trigger the NLRP3 inflammasome, promoting the maturation of IL-1β/18. Created with BioGDP.com [76].

In terms of self-antigen production, retrotransposon activity generates a variety of immunogenic substances. Their nucleic acid components can be recognized by autoantibodies, forming pathogenic immune complexes; encoded proteins such as ORF1p can be detected in patients and can activate autoreactive immune cells [74]. More notably, transposon-derived peptide segments may induce cross-immunity reactions through molecular simulation mechanisms. These mechanisms work together to disrupt the immune system’s self-tolerance, creating a vicious cycle in autoimmune diseases such as SLE, leading to the continuous production of autoantibodies and the exacerbation of inflammatory reactions (Figure 2) [75].

**Figure 3:**
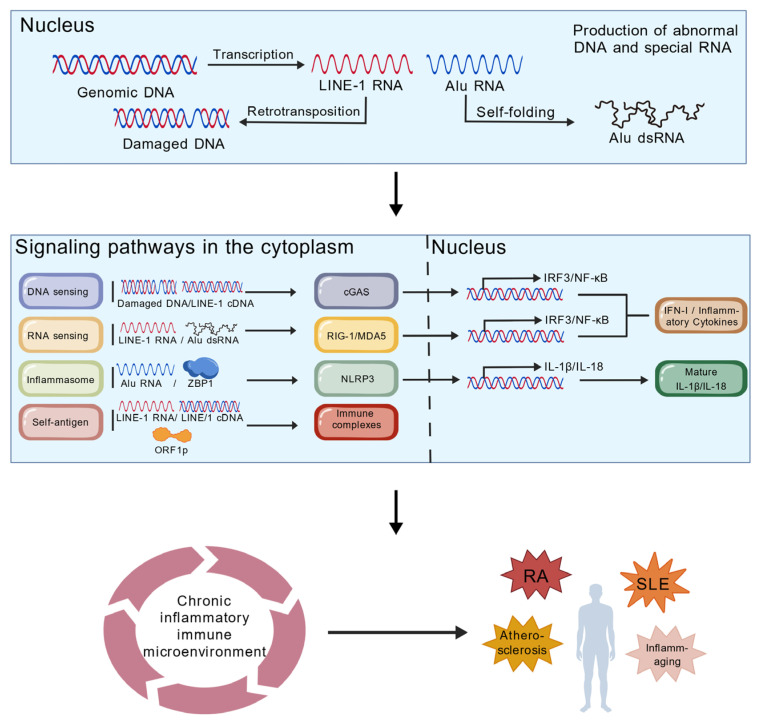
Mechanism of retrotransposon-induced chronic inflammation and autoimmunity [77]. Genomic disruption leads to their transcription, producing LINE-1 RNA and Alu dsRNA. LINE-1 generates cDNA via transposition and causes DNA damage. These nucleic acids activate distinct cytoplasmic pathways: cGAS-STING and RIG-I/MDA5 pathways produce type I interferons and inflammatory factors; NLRP3 inflammasome processes and releases IL-1β/IL-18; Autoimmune responses generate autoantibodies targeting LINE-1 cDNA, LINE-1 RNA, and ORF1p as antigens. All outputs synergistically shape a self-sustaining chronic inflammatory immune microenvironment locally, ultimately leading to diseases such as systemic lupus erythematosus (SLE), rheumatoid arthritis (RA), atherosclerosis, and age-related inflammation. Created with BioGDP.com [76].

Overall, LINE and SINE retrotransposons form a complete pathogenic network in sterile inflammatory diseases through three main pathways, DNA sensing, RNA sensing, and inflammasome activation, as well as the production of autoantigens. These mechanisms work together and reinforce each other: the nucleic acid sensing pathway establishes the inflammatory foundation, the inflammasome amplifies the inflammatory response, and the production of autoantigens leads to the persistence and autoimmunization of the inflammatory response. This multi-layered activation pattern not only explains why retrotransposon activation leads to such intense inflammatory responses but also provides multiple potential intervention targets for developing novel therapeutic strategies. Future research needs to further elucidate the relative importance of these pathways in different diseases and their interplay, laying the theoretical foundation for precision medicine (Figure 3) [77].

## 7. The Therapeutic Potential of LINEs and SINEs in Sterile Inflammatory Diseases

The therapeutic potential of targeting non-LTR retrotransposons is currently being evaluated on two primary fronts: upstream strategies that directly suppress their activity [78] and downstream approaches that regulate the inflammatory pathways they trigger [79]. Breakthrough progress has been made in developing candidate drugs that target LINE-1 reverse transcriptase [80]. For instance, the oral inhibitor TPN-101 demonstrated efficacy in Phase 2 clinical trials for PSP and ALS by reducing neuroinflammation-related biomarkers and stabilizing functional metrics in patients. Having secured FDA Fast Track designation, this drug signals that therapeutic approaches regulating sterile inflammation by inhibiting retrotransposon activity represent a highly promising new direction in drug development. In the context of Aicardi–Goutières syndrome (AGS), a classic inherited sterile inflammation disorder, a Phase 2a clinical trial has also been initiated investigating TPN-101 for AGS patients. This study aims to evaluate whether TPN-101 can safely and effectively reduce excessive interferon signaling in patients by inhibiting the reverse transcription activity of retrotransposons, thereby diminishing their inflammatory nucleic acid products at the source (Table 1) [81]. Meanwhile, a Phase 1 clinical trial (LINE-AD trial) is underway to evaluate the use of the classic HIV reverse transcriptase inhibitor emtricitabine for treating Alzheimer’s disease, based on the hypothesis that it inhibits age-related LINE-1 activation in the brain. Together, these two cases demonstrate the transformative potential of intervening in neuroinflammation and systemic sterile inflammation by suppressing reverse transcriptase activity (Table 1) [82].

Recent studies have shown that active retrotransposition of LINEs and SINEs is closely related to the pathogenesis of sterile inflammatory diseases, providing new possibilities for the development of targeted treatment strategies [83]. A paper published in Nature in 2019 found that LINE-1 is closely associated with age-related inflammation and aging-related secretory phenotypes, it also mentioned that the HIV reverse transcriptase inhibitor lamivudine has LINE-1 RT inhibitory activity, which can downregulate interferon (IFN-I) activation and age-related inflammation in aging mice, representing the first discovery of a LINE-1 inhibitor [61]. However, reverse transcriptase inhibitors (e.g., lamivudine) only inhibit cDNA synthesis and cannot block Alu double-stranded RNA-mediated inflammatory responses. MyD88 (myeloid differentiation factor 88) is a key adaptor protein that plays a pivotal role in immune signaling pathways. It primarily mediates the signaling pathways of the TLR and interleukin-1 receptor (IL-1R) families. Related studies have shown that MyD88 inhibitors can block Alu RNA-induced retinal pigment epithelial cell degeneration by inhibiting the MyD88-mediated inflammatory signaling pathway, providing a potential new target and therapeutic strategy for the treatment of age-related macular degeneration (AMD) [84]. The application of small molecule inhibitors, such as drug inhibitors of histone demethylase KDM4B, can significantly reduce the expression of LINE-1 in breast cancer cells, identifying novel regulatory factors that may effectively treat sterile inflammatory diseases by regulating metabolic pathways associated with LINEs and SINEs [85]. Additionally, studies have revealed that nuclear cGAS restricts LINE-1 retrotransposition activity by promoting TRIM41-mediated ubiquitination and degradation of the ORF2p protein, thereby maintaining genomic stability. Based on the regulatory mechanism of nuclear cGAS on LINE-1 activity, new small-molecule inhibitors can be developed to modulate nuclear cGAS activity, thereby controlling LINE-1 retrotransposition, as well as the development of cGAS and STING inhibitors [86].

Nuclearly localized TDP-43 is a key epigenetic regulator maintaining LINE-1 transposon silencing. In the brains of patients with frontotemporal dementia–amyotrophic lateral sclerosis (FTD-ALS), pathological nuclear TDP-3 loss (accompanied by abnormal cytoplasmic aggregation) directly drives chromatin decondensation at LINE1 sites. This leads to increased LINE-1 DNA content and enhanced transposon activity, promoting genomic instability. Notably, antiretroviral drugs effectively suppress TDP-43-deficient LINE1 transposon activation, providing a novel potential target for targeted intervention in TDP-43-related proteinopathies [87]. Research has shown that RNA editing via ADAR1 can prevent MDA5 from abnormally activating endogenous Alu RNA as its ligand, thereby preventing the onset of a series of sterile inflammatory diseases. This can prevent the activation of endogenous transcripts in the cytoplasmic dsRNA response, thereby reducing abnormal activation of the immune system. This approach holds promise as a new strategy for treating sterile inflammatory diseases associated with LINEs and SINEs [88]. However, targeted modulation of the ADAR1 and cGAS pathways carries risks, as both overactivation and suppression can drive disease progression [78]. Active LINEs and SINEs can serve as biomarkers for diagnosing and monitoring the progression of sterile inflammatory diseases. For example, studies have shown that LINE-1 methylation levels can act as a new epigenetic biomarker for predicting the response of patients with early rheumatoid arthritis (ERA) who are methotrexate (MTX)-responsive but seronegative. By detecting the methylation of LINEs and SINEs, treatment regimens can be adjusted in a timely manner to enhance treatment efficacy [45]. Additionally, RNA polymerase III inhibitors, targeted epigenetic modulators, RNA degradation, and other approaches may also hold significant therapeutic potential in sterile inflammatory diseases [89]. Future research will further reveal the specific mechanisms of action of LINEs and SINEs in sterile inflammatory diseases. Through the comprehensive application of multiple mechanisms and strategies, this research will lay the foundation for the development of new treatment methods [90].

## 8. Conclusions

Abnormal activity of LINEs and SINEs is closely associated with various sterile inflammatory diseases, directly or indirectly contributing to their onset and progression, indicating their significant physiological and pathological roles in such diseases. Its mechanism is not only reflected in the complete pathogenic network formed by the three pathways of DNA sensing, RNA sensing, and inflammasome activation, as well as the generation of autoantigens, but also includes genomic instability, epigenetic regulation, and inflammation. This indicates its important physiological and pathological roles in non-infectious inflammatory diseases. In-depth research into the role of LINEs and SINEs in sterile inflammatory diseases not only helps elucidate the pathogenesis of these diseases but may also provide new targets for diagnosis and treatment. Future studies should further explore the specific mechanisms of LINEs and SINEs in sterile inflammatory diseases and develop novel therapeutic strategies targeting retrotransposon activity.

## Figures and Tables

**Table 1:** Clinical research progress on non-LTR retrotransposon inhibitors [81,82].

Drug Name	Indications	Trial Phase	Test Number
Emtricitabine	Alzheimer’s disease	Phase I	NCT04500847
TPN-101	AGS	Phase II	NCT05613868
TPN-101	Progressive Supranuclear Palsy (PSP)	Phase II	NCT04993768
TPN-101	Amyotrophic lateral Sclerosis (ALS)	Phase II	NCT04993755

## Data Availability

No new data were created or analyzed in this study. Data sharing is not applicable to this article.

## Abbreviations

The following abbreviations are used in this manuscript:

AGS	Aicardi–Goutières Syndrome
AMD	Age-Related Macular Degeneration
DAMPs	Damage-Associated Molecular Patterns
LINE	Long Interspersed Nuclear Element
LTR	Long Terminal Repeat
MS	Multiple Sclerosis
SINE	Short Interspersed Nuclear Element
SLE	Systemic Lupus Erythematosus
SS	Sjögren’s Syndrome
TPRT	Target-Primed Reverse Transcription
